# Organic Peroxide-Sensing Repressor OhrR Regulates Organic Hydroperoxide Stress Resistance and Avermectin Production in *Streptomyces avermitilis*

**DOI:** 10.3389/fmicb.2018.01398

**Published:** 2018-06-29

**Authors:** Meng Sun, Mengya Lyu, Ying Wen, Yuan Song, Jilun Li, Zhi Chen

**Affiliations:** State Key Laboratory of Agrobiotechnology and Key Laboratory of Soil Microbiology, Ministry of Agriculture, College of Biological Sciences, China Agricultural University, Beijing, China

**Keywords:** *Streptomyces avermitilis*, avermectin biosynthesis, OhrR, organic hydroperoxide stress resistance, repression

## Abstract

The bacterium *Streptomyces avermitilis* is an industrial-scale producer of avermectins, which are important anthelmintic agents widely used in agriculture, veterinary medicine, and human medicine. During the avermectin fermentation process, *S. avermitilis* is exposed to organic peroxides generated by aerobic respiration. We investigated the role of MarR-family transcriptional regulator OhrR in oxidative stress response and avermectin production in *S. avermitilis*. The *S. avermitilis* genome encodes two organic hydroperoxide resistance proteins: OhrB1 and OhrB2. OhrB2 is the major resistance protein in organic peroxide stress responses. In the absence of organic peroxide, the reduced form of OhrR represses the expression of *ohrB2* gene by binding to the OhrR box in the promoter region. In the presence of organic peroxide, the oxidized form of OhrR dissociates from the OhrR box and the expression of *ohrB2* is increased by derepression. OhrR also acts as a repressor to regulate its own expression. An *ohrR*-deletion mutant (termed DohrR) displayed enhanced avermectin production. Our findings demonstrate that OhrR in *S. avermitilis* represses avermectin production by regulating the expression of pathway-specific regulatory gene *aveR*. OhrR also plays a regulatory role in glycolysis and the pentose phosphate (PP) pathway by negatively controlling the expression of *pykA2* and *ctaB*/*tkt2*-*tal2*-*zwf2*-*opcA2*-*pgl*.

## Introduction

The organic peroxide-sensing repressor OhrR is a MarR family transcriptional regulator that regulates the expression of organic hydroperoxide resistance protein (Ohr) (Fuangthong et al., 2001; Sukchawalit et al., 2001). OhrR is widely distributed in Gram-positive and Gram-negative bacteria (Chuchue et al., 2006; Oh et al., 2007; Atichartpongkul et al., 2010; da Silva Neto et al., 2012; Saikolappan et al., 2015). Most *ohrR* genes are genetically linked to *ohr* genes, either expressed divergently from a bidirectional promoter region (Chuchue et al., 2006; Oh et al., 2007; Saikolappan et al., 2015) or co-transcribed in an operon (Sukchawalit et al., 2001; Atichartpongkul et al., 2010).

The DNA-binding sequence of OhrR (OhrR box) has been identified in some bacteria as a conserved AT-rich inverted repeat region. Examples of OhrR box consensus sequences are TACAATTNAATTGTA in *Bacillus subtilis* (Fuangthong and Helmann, 2002), TTnCAATT-(16/17)-AATTGnAA in *Xanthomonas campestris* (Mongkolsuk et al., 2002), TACAATTNAATTGTA in *Agrobacterium tumefaciens* (Chuchue et al., 2006), and GCAACTNAATTGC in *Streptomyces coelicolor* (Oh et al., 2007). In the absence of organic peroxide, the reduced form of OhrR binds to the OhrR box in the *ohr* promoter region and represses *ohr* expression (Panmanee et al., 2002; Chuchue et al., 2006; Oh et al., 2007; Saikolappan et al., 2015). Upon exposure to organic hydroperoxides, oxidation of the conserved cysteine (Cys) residue in the OhrR N-terminus leads to a major conformational change of OhrR, resulting in DNA dissociation and allowing RNA polymerase to bind to the promoter and initiate *ohr* gene transcription (Hong et al., 2005; Lee et al., 2007; Newberry et al., 2007; Soonsanga et al., 2007).

OhrRs are divided into two classes (single-Cys and multiple-Cys) according to presence vs. absence of additional C-terminal Cys residues (Panmanee et al., 2006). OhrRs from *B. subtilis* and *S. coelicolor* have a single N-terminal Cys residue (Hong et al., 2005; Oh et al., 2007). OhrRs from many other species, including *X. campestris*, *A. tumefaciens*, and *Pseudomonas aeruginosa*, have two or more Cys residues (Chuchue et al., 2006; Newberry et al., 2007; Atichartpongkul et al., 2010; Atichartpongkul et al., 2016). In *B. subtilis*, oxidation of the single Cys residue (Cys^15^) results in a sulfenic acid intermediate (Fuangthong and Helmann, 2002; Hong et al., 2005), which is further oxidized to generate mixed disulfides or a protein sulfenamide derivative, with consequent dissociation of OhrR from operator DNA (Lee et al., 2007). In *X. campestris*, upon organic hydroperoxide oxidation, the N-terminal Cys residue (Cys^22^) of OhrR forms a reversible intersubunit disulfide bond with C-terminal Cys^127′^ between the two subunits of OhrR dimer. This conformational change results in 28° rotation of each winged helix-turn-helix, and DNA dissociation (Panmanee et al., 2006; Newberry et al., 2007).

Soil-dwelling *Streptomyces* bacteria are characterized by complex morphological differentiation and secondary metabolism. To avoid harmful effects of various oxidants present in the environment or generated by aerobic metabolism, *Streptomyces* utilize specific antioxidant enzymes, notably alkyl hydroperoxide reductase (AhpCD), catalase (CatA), Ohr, and thioredoxin systems, which are mediated, respectively, by transcriptional regulators OxyR (Hahn et al., 2002; Liu et al., 2016), CatR (Hahn et al., 2000), OhrR (Oh et al., 2007), and σ^R^ (Kim et al., 2012). Upon exposure to organic hydroperoxides in *S. coelicolor*, OhrR acts as a repressor of *ohrA* and an activator of its own gene (Oh et al., 2007).

*S. avermitilis* is an industrial-scale producer of avermectins, a series of 16-membered macrocyclic lactone derivatives widely used as drugs or pesticides in agriculture, veterinary medicine, and human medicine (Ikeda and Omura, 1997). During the avermectin fermentation process, *S. avermitilis* is exposed to organic peroxides generated by aerobic respiration. We investigated the regulatory role of OhrR in oxidative stress response and avermectin production in *S. avermitilis*.

## Materials and Methods

### Strains, Plasmids, Culture Conditions, and Culture Media

Strains and plasmids used in this study are listed in **Table 1**. Culture conditions for *S. avermitilis* sporulation, protoplast preparation, and regeneration were as described previously (Macneil and Klapko, 1987). Seed medium and fermentation medium FM-I were used for avermectin production and for RNA isolation (Jiang et al., 2011). YEME were also used for RNA isolation with peroxide treatment. *E. coli* strains were grown at 37°C in LB medium.

**Table 1:** Strains and plasmids used in this study.

Strain or plasmid	Description	Source
*S. avermitilis*		
ATCC31267	Wild-type strain (WT)	Laboratory stock
DohrR	*ohrR* deletion mutant	This study
CohrR	*ohrR* complementation strain	This study
*E. coli*		
JM109	General cloning host	Laboratory stock
BL21 (DE3)	Host for protein overexpression	Laboratory stock
Plasmids		
pKC1139	Multiple-copy, temperature-sensitive *E. coli*–*Streptomyces* shuttle vector	Bierman et al., 1992
pSET152	Integrative *E. coli*–*Streptomyces* shuttle vector	Bierman et al., 1992
pET-28a (+)	Vector for His_6_-tagged protein overexpression in *E. coli*	Novagen
pKCD-ohrR	*ohrR* deletion vector based on pKC1139	This study
pSET-ohrR	*ohrR* complementation vector based on pSET152	This study
pET-OhrR	*ohrR* overexpression vector based on pET-28a (+)	This study


### Gene Deletion and Complementation

An *ohrR* (*SAV5090*) gene deletion mutant was constructed using traditional homologous recombination strategy. Two DNA fragments flanking *ohrR* gene were amplified by PCR from *S. avermitilis* ATCC31267 genomic DNA. A 510-bp fragment upstream of *ohrR* (position -426 to +84 from start codon) was amplified by primers OR-up-Fw and OR-up-Rev, and a 431-bp fragment downstream of *ohrR* (position +445 to +875) was amplified by primers OR-dw-Fw and OR-dw-Rev (Supplementary Table S1 and Supplementary Figure S1). The two fragments were recovered, digested, respectively, by *Eco*RI/*Xba*I and *Bam*HI/*Xba*I, and ligated together into pKC1139 (Bierman et al., 1992) to produce *ohrR*-deletion vector pKCD-ohrR. pKCD-ohrR was introduced into ATCC31267 protoplasts, and double-crossover mutants were selected as described previously (Zhao et al., 2007). The *ohrR*-deletion mutant (termed DohrR) was confirmed by PCR using OR-V-Fw/OR-V-Rev (external primers) and OR-V2-Fw/OR-V2-Rev (internal primers), followed by DNA sequencing (Supplementary Table S1 and Supplementary Figure S1).

For complementation analysis of DohrR, *ohrR* ORF and its promoter were amplified by PCR using primers OR-C-Fw and OR-C-Rev (Supplementary Table S1), and ligated into *Bam*HI/*Eco*RI-digested pSET152 to produce complementation vector pSET-ohrR. The resulting pSET-ohrR was transformed into DohrR protoplasts to produce a complementation strain of DohrR (termed CohrR).

### RNA Preparation and Quantitative Real-Time RT-PCR Analysis (qRT-PCR)

RNA was isolated from *S. avermitilis* mycelia grown in FM-I or YEME, using TRIzol reagent (Tiangen, China) as described previously (Guo et al., 2013). The chromosomal DNA contamination of RNA samples was removed by adding DNase I (TaKaRa, Japan). The concentrations of RNA were measured by NanoVue Plus spectrophotometer (GE Healthcare). Each RNA sample (2 μg) was reverse transcribed by random hexamers (25 μM), dNTP mixture (10 mM) and M-MLV (TaKaRa, Japan). qRT-PCR was performed to determine the transcription levels of various genes using primers listed in Supplementary Table S1. Each reaction system (20 μl) contains template cDNA, forward and reverse primers (each 300 nM) and 10 μl FastStart Universal SYBR Green Master (ROX). PCR protocol: 95°C for 10 min, 40 cycles of 95°C for 10 s/60°C for 30 s. The relative expression level was calculated using the comparative Ct method. *hrdB* gene was used as internal control.

### Fermentation and HPLC Analysis of Avermectin Production

Fermentation of *S. avermitilis* strains and HPLC analysis of avermectin production were performed as described previously (Jiang et al., 2011).

### Overexpression and Purification of His_6_-OhrR

For overexpression of the N-terminal His_6_-tagged OhrR in *E. coli*, *ohrR* ORF was amplified using primers His-ohrR-Fw and His-ohrR-Rev (Supplementary Table S1). The DNA fragment was purified, cut with *Eco*RI/*Hin*dIII, and cloned into pET28a (+) to generate the expression plasmid pET-OhrR, which was then introduced into *E. coli* BL21 (DE3) for overexpression. His_6_-OhrR was induced by treatment with 0.2 mM IPTG for 12 h at 16°C. Cells were collected and disrupted in lysis buffer (20 mM Tris base, 500 mM NaCl, 5 mM imidazole, 5% glycerol [pH 7.9]) by sonication on ice. His_6_-OhrR was purified from the lysate using Ni^2+^-NTA resin (Bio-works, Sweden) as per the manufacturer’s protocol.

### Electrophoretic Mobility Gel Shift Assays (EMSAs)

Electrophoretic mobility gel shift assays (EMSAs) were performed using a DIG Gel Shift Kit (2nd Generation, Roche) as per the manufacturer’s protocol. DNA probes were amplified by PCR (Supplementary Table S1) and labeled with digoxigenin using recombinant terminal transferase. Binding reactions and detection conditions were as described previously (Liu et al., 2013). Various concentrations of tert-butyl hydroperoxide (*t*BHP) and dithiothreitol (DTT) were added to the mixture to evaluate the effects of oxidation on OhrR binding activity.

### Determination of Transcriptional Start Sites (TSSs)

The TSSs of *ohrR* and *ohrB2* were analyzed using a 5′/3′ RACE Kit (2nd Generation, Roche) as per the manufacturer’s protocol. Total RNA of wild-type strain ATCC31267 (WT) grown in FM-I for 6 days was used as the template. Primers used are listed in Supplementary Table S1.

### DNase I Footprinting Assays

A fluorescence labeling method was used for these assays. DNA probes were obtained by PCR using FAM-labeled primers (Supplementary Table S1). Reaction mixtures (each 20 μL) containing labeled DNA fragments and various quantities of His_6_-OhrR were incubated for 30 min at 25°C. DNase I digestion was performed for 40 s at 37°C, and stopped by addition of 60 μM EDTA (pH 8.0). After phenol/chloroform extraction and ethanol precipitation, the samples were subjected to capillary electrophoresis. Data were analyzed using GeneMarker software v2.2.0.

## Results

### *ohrB2* Expression Is Upregulated in DohrR

The annotated *S. avermitilis* genome revealed one *ohrR* gene (*SAV5090*) and two *ohr* genes, *ohrB1* (*SAV3610*), and *ohrB2* (*SAV5091*). *ohrR* and *ohrB2* are transcribed from a bidirectional promoter region. OhrB1 and OhrB2 contain two highly conserved Cys residues, similar to Ohr proteins in other bacteria (Atichartpongkul et al., 2001). In contrast to *S. coelicolor* OhrR, which has a single Cys residue (Oh et al., 2007), *S. avermitilis* OhrR has a C-terminal Cys residue (Cys^164^) in addition to the N-terminal conserved Cys residue (Cys^25^).

**FIGURE 1:**
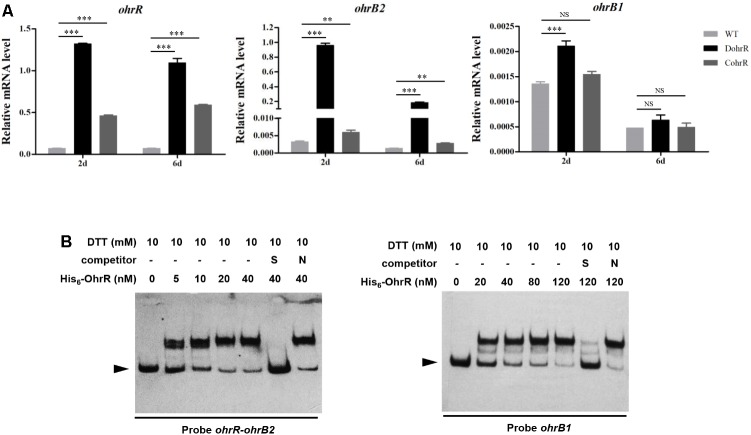
Interaction of OhrR with *ohrR–ohrB2* intergenic region and *ohrB1* promoter. **(A)** qRT-PCR of *ohr* expression in DohrR, CohrR and WT. RNA was prepared from cells grown in FM-I for 2 or 6 days. Values shown are mean ± SD from three technical replicates. ^∗∗^*P* < 0.01; ^∗∗∗^*P* < 0.001; NS, not significant (Student’s *t*-test). **(B)** EMSAs of His_6_-OhrR with *ohrR–ohrB2* intergenic region and *ohrB1* promoter region. Competition assays were performed using 200-fold excess of specific (S) and non-specific (N) unlabeled DNAs. Top: concentrations of His_6_-OhrR and DTT. Bottom: probes used. Arrow: free probe.

An *ohrR*-deletion mutant, DohrR, was constructed by homologous recombination as described in the section “Materials and Methods.” *ohrR* deletion had no effect on morphology, but slightly inhibited growth of *S. avermitilis*, and the decreased growth could be complemented by introduction of a single-copy *ohrR* gene to DohrR (Supplementary Figure S1). Sensitivity of DohrR to oxidants (0.2 mM *t*BHP or 1 mM H_2_O_2_) was the same as that of WT (Supplementary Figure S2). Transcription levels of *ohrR*, *ohrB2*, and *ohrB1* in FM-I were analyzed by RT-qPCR for ATCC31267 and DohrR. *ohrB2* expression in DohrR was much higher than in WT, increased by approximately 300-fold on day 2 (exponential phase) and 135-fold on day 6 (stationary phase), indicating a negative role of OhrR in regulation of *ohrB2* expression (**Figure 1A**). *ohrR* transcription levels were increased by 18-fold on day 2 and 15-fold on day 6 in DohrR than in WT, indicating that OhrR also negatively regulates its own expression. *ohrB1* transcription levels in DohrR were only slightly higher than those in WT, suggesting that *ohrB1* is not tightly controlled by OhrR. The transcriptional levels of *ohrB2* and *ohrB1* were restored to WT level in CohrR strain, indicating that OhrR is responsible for the observed effects on transcription. However, the expression of *ohrR* was only partially complemented in CohrR strain, still higher than that of WT, probably because *ohrR* gene only functions properly *in cis*.

### Determination of OhrR Binding Site of *ohrR*–*ohrB2* Intergenic Region

OhrR protein was overexpressed in *E. coli* with an N-terminal His_6_-tag, and purified for EMSAs. His_6_-OhrR bound specifically to the *ohrR*–*ohrB2* intergenic region and the promoter region of *ohrB1* (**Figure 1B**). The quantity of His_6_-OhrR required to generate intense shifted bands was greater for *ohrB1* promoter than for *ohrR*–*ohrB2* intergenic region, suggesting that OhrR interacts more weakly with *ohrB1* promoter.

**FIGURE 2:**
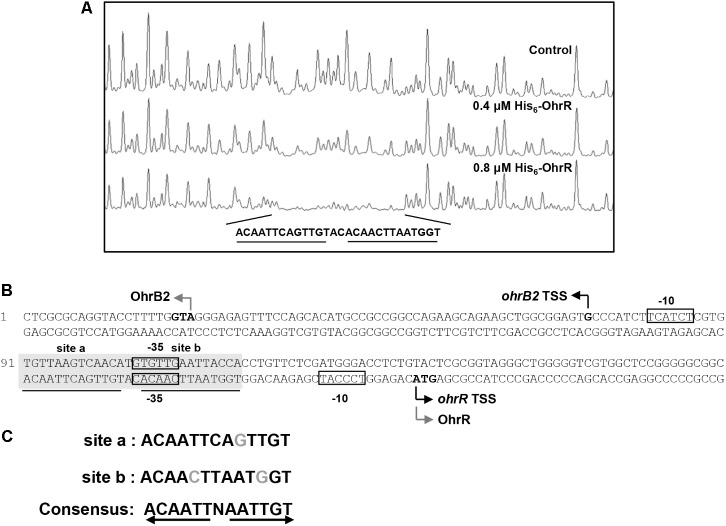
DNase I footprinting assay of *ohrR–ohrB2* intergenic region using His_6_-OhrR. **(A)** Fluorograms corresponding to control DNA and to protected reactions with 0.4 and 0.8 μM His_6_-OhrR. **(B)** Nucleotide sequences of *ohrR–ohrB2* intergenic region. Non-shaded boxes: presumed –35 and –10 regions of *ohrR* and *ohrB2*. Shaded boxes: regions protected by His_6_-OhrR. Underlining: OhrR motif (site a and site b). Gray bent arrows: translational start codons. Black bent arrows: TSSs. **(C)** Consensus sequence of OhrR motif.

DNase I footprinting assay was performed to detect the OhrR binding site in the *ohrR*–*ohrB2* intergenic region. A 28-nt protected region was detected in the presence of 0.4 μM and 0.8 μM His_6_-OhrR (**Figure 2A**). Two similar 13-nt sites (ACAATTCAGTTGT [site a], ACAACTTAATGGT [site b]) were found in the protected region (**Figure 2B**). The consensus sequence of OhrR box (ACAATTNAATTGT) in *S. avermitilis* (**Figure 2C**) was similar to that of OhrR box (GCAACTNAATTGC) in *S. coelicolor* (Oh et al., 2007). Identified OhrR boxes extend from positions -45 to -18 relative to TSS for *ohrB2*, and from positions -50 to -23 relative to TSS for *ohrR*. In both cases, the protected region is located in the promoter and contains the -35 region. OhrR Thus represses *ohrB2* and *ohrR* transcription by blocking attachment of RNA polymerase to its promoter.

### OhrR Mediates Organic Peroxide Induction of *ohrB2* by Derepression

**FIGURE 3:**
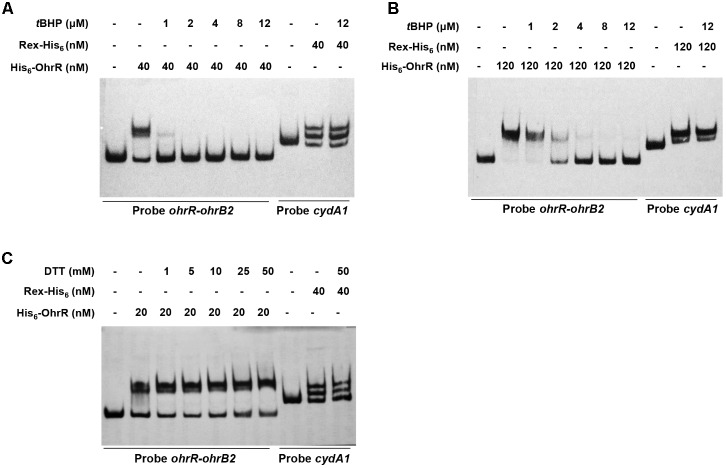
Effects of *t*BHP **(A,B)** and DTT **(C)** on *in vitro* binding of His_6_-OhrR to *ohrR–ohrB2* intergenic region. Concentrations of His-OhrR, *t*BHP, and DTT are shown at top. Rex-His_6_ was used as DNA binding protein control.

Effects of OhrR oxidation on binding affinity to target DNA were evaluated by adding various concentrations of *t*BHP and DTT to EMSA mixtures. *t*BHP inhibited DNA binding between His_6_-OhrR and the *ohrR*–*ohrB2* intergenic region at very low concentration. When low concentration (40 nM) of His_6_-OhrR was used for EMSA, 1 μM *t*BHP was sufficient to eliminate binding, whereas when high concentration (120 nM) of His_6_-OhrR was used, 2 μM *t*BHP was required to eliminate binding (**Figures 3A,B**). *t*BHP had no effect on binding affinity of control DNA binding protein Rex to its target promoter *cydA1*, suggesting that OhrR specifically senses and responds to organic oxidant *t*BHP. In DTT experiments, binding increased slightly as DTT concentration increased, indicating that interaction with target DNA was stronger for reduced than for oxidized His_6_-OhrR (**Figure 3C**). The enhancing effect of DTT on binding affinity was more noticeable when the interaction was weak or when lower OhrR concentration was used for EMSA (Supplementary Figure S3).

**FIGURE 4:**
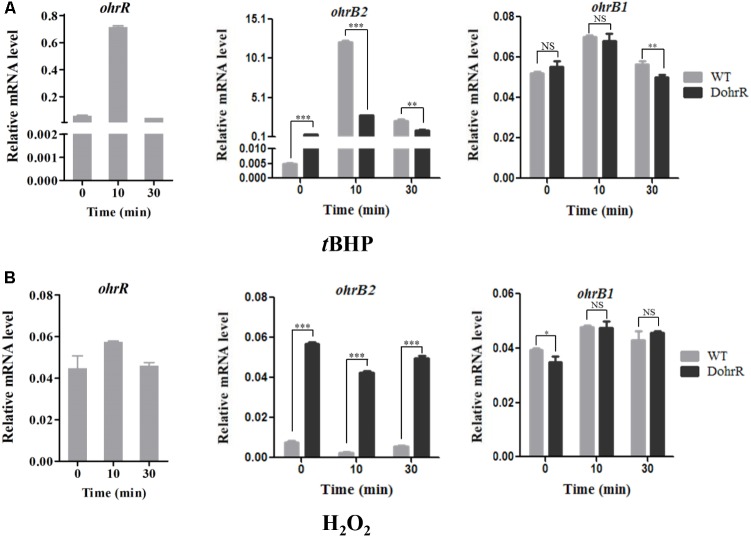
qRT-PCR of oxidant-induced genes in WT and DohrR. RNA was prepared from cells grown in YEME and treated with 0.4 mM *t*BHP **(A)** or 1 mM H_2_O_2_ **(B)** for 10 or 30 min. No peroxide was added at 0 min. Values shown are mean ± SD from three replicates. ^∗^*P* < 0.05; ^∗∗^*P* < 0.01; ^∗∗∗^*P* < 0.001; NS, not significant (Student’s *t*-test).

*ohr* expression levels in peroxide-treated DohrR and WT were analyzed by qRT-PCR. *S. avermitilis* cells were cultured in YEME for 42 h and 0.4 mM *t*BHP or 1 mM H_2_O_2_ was added. Cells were treated for 10 or 30 min. Compared to untreated (0 min), 10 min *t*BHP treatment increased *ohrR* transcription to a maximal level in WT, and H_2_O_2_ treatment did not induce *ohrR* transcription in WT (**Figure 4**), indicating that OhrR responds specifically to organic peroxides. The transcription level of *ohrB2* in WT increased by >1000-fold to its maximal value within 10 min of *t*BHP treatment, and decreased gradually with extended treatment. The transcription profile of *ohrB2* in DohrR was similar to that in WT under *t*BHP treatment condition, only with a much lower increased fold at its maximal value. *ohrB2* transcription was not induced by H_2_O_2_ treatments in both strains, but were much higher in untreated DohrR than in untreated WT. Thus, OhrR negatively regulated *ohrB2* expression in the absence of peroxide, whereas in the presence of peroxide, OhrR interaction with *ohrB2* promoter was eliminated and *ohrB2* expression was improved by derepression. *ohrB1* expression was only slightly induced by *t*BHP treatment in WT, and its expression was much lower than that of *ohrB2* under *t*BHP treatment condition, indicating that OhrB2 is the major organic hydroperoxide resistance protein in *S. avermitilis*. *ohrB1* expression was also slightly induced by H_2_O_2_ treatment, and *ohrB1* levels under both treatments were similar for DohrR and WT, suggesting that the slight induction of *ohrB1* by either treatment is not mediated by OhrR.

### OhrR Negatively Regulates Avermectin Production

**FIGURE 5:**
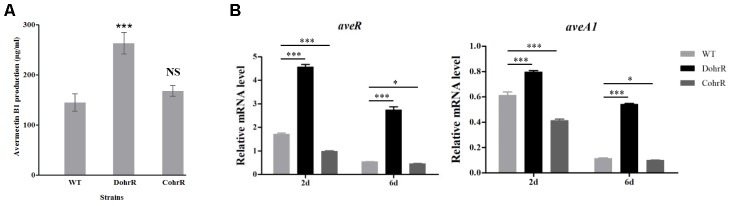
Effect of *ohrR* deletion on avermectin production. **(A)** Avermectin production in *ohrR*-related mutants. **(B)** qRT-PCR of *aveR* and *aveA1* transcription levels in WT, DohrR and CohrR. RNA samples as in **Figure 1**. ^∗^*P* < 0.05; ^∗∗∗^*P* < 0.001; NS, not significant (Student’s *t*-test).

*ohrR* deletion (DohrR mutant) caused a ˜2-fold increase in avermectin production (**Figure 5A**). Avermectin production was restored to WT level by re-introduction of *ohrR* gene to DohrR, indicating a negative regulatory role of OhrR in the process. To examine the causes of avermectin overproduction in DohrR, we measured the transcription levels of selected avermectin biosynthesis-related genes (pathway-specific regulatory gene *aveR* and biosynthetic gene *aveA1*) in DohrR and WT by qRT-PCR. RNA samples were prepared from cells grown in FM-I for 2 and 6 days. Transcription levels of *aveR* and *aveA1* were higher in DohrR than in WT on both day 2 and day 6, and increased by >4-fold at day 6 (**Figure 5B**). The transcription levels of both genes were restored to WT levels in CohrR. These findings indicate that *ave* expression was repressed by OhrR, especially at the late stage of avermectin production.

### OhrR Binds to *aveR* Promoter Region

**FIGURE 6:**
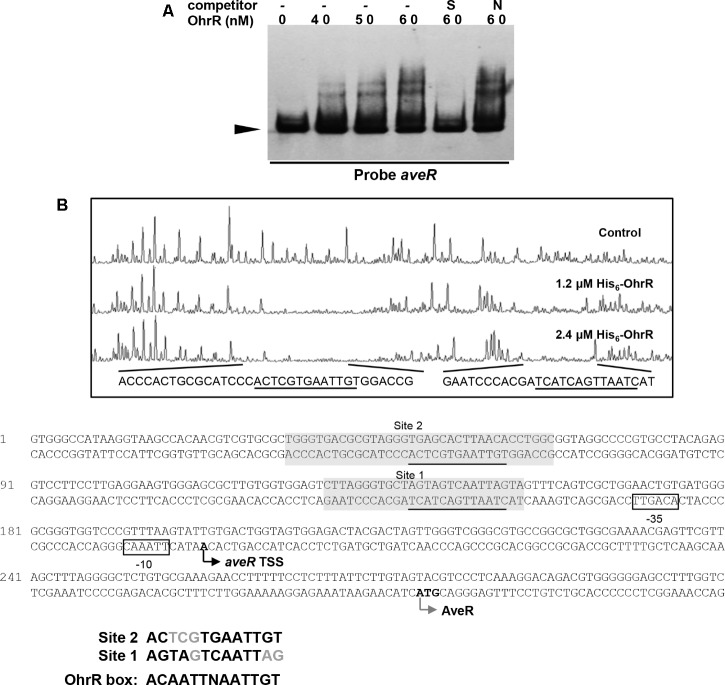
Binding of OhrR to *aveR* promoter region. **(A)** EMSA of His_6_-OhrR with *aveR* promoter region. **(B)** DNase I footprinting assay of *aveR* promoter region using His_6_-OhrR and nucleotide sequences of *ohrR–ohrB2* intergenic region. EMSA and DNase I footprinting conditions as in **Figures 1, 2**.

Electrophoretic mobility gel shift assays were performed using the promoter regions of *ave* genes and His_6_-OhrR to determine whether OhrR directly regulates transcription of the genes. With the *aveR* promoter region and His_6_-OhrR, two retarded bands were observed. The shifted bands were completed abolished by addition of unlabeled specific probe, but not by non-specific competitor probe (**Figure 6A**), indicating that the interaction between OhrR and the *aveR* promoter region was specific. With the *aveA1* promoter region, no retarded band was observed (data not shown), indicating that OhrR represses avermectin production by regulating the expression of regulatory gene *aveR*.

To determine the OhrR binding site in the *aveR* promoter region, DNase I footprinting assays were performed using fluorescein-labeled *aveR* promoter region (position -426 to +51 from start codon) and His_6_-OhrR. In the presence of 1.2 or 2.4 μM His_6_-OhrR, two protected regions were observed, one extending 26 nt (site 1) and the other 35 nt (site 2) (**Figure 6B**), consistently with the two retarded bands observed in EMSA (**Figure 6A**). Two possible 11-nt OhrR binding sites (AGTAGTCAATTAG in site 1; ACTCGTGAATTGT in site 2) were found in the protected regions. The OhrR boxes are, respectively, located from positions -153 to -141 and -63 to -51 relative to the TSS of *aveR* (Zhuo et al., 2010). These findings, in combination with transcription results, suggest that OhrR possibly represses the transcription of *aveR* by impeding attachment of RNA polymerase to the promoters.

### OhrR Binds to *pykA2* and *tkt2*/*ctaB* Promoter Regions

**FIGURE 7:**
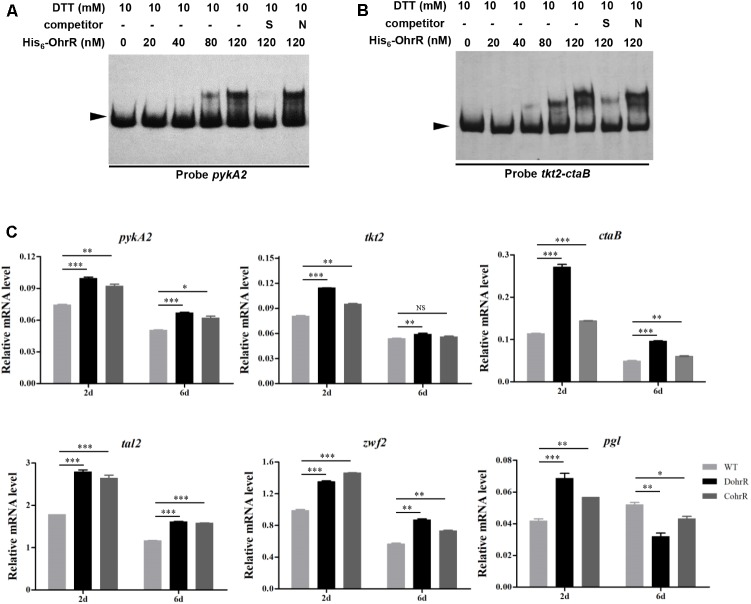
Binding of His_6_-OhrR to *pykA2* promoter region **(A)** and *tkt2-ctaB* intergenic region **(B)**. EMSAs using His_6_-OhrR protein at the indicated concentrations. EMSA conditions as in **Figure 1**. **(C)** qRT-PCR of *pykA2*, *tkt2, ctaB*, *tal2, zwf2,* and *pgl* transcription levels in WT, DohrR, and CohrR. ^∗^*P* < 0.05; ^∗∗^*P* < 0.01; ^∗∗∗^*P* < 0.001; NS, not significant (Student’s *t*-test).

In a search for additional putative OhrR targets in *S. avermitilis*, we scanned the *S. avermitilis* genome with the consensus sequence ACAATTNAATTGT by Virtual Footprint program (Munch et al., 2005). We found one annotated gene (*pykA2*) with two mismatches, and nine genes (*sig7*, *aveR*, *ilvA*, *ohrB1*, *cspD3*, *paaA*, *tkt2*/*ctaB*, *fadE3*, *sig57*) with three mismatches. In view of our finding that OhrR directly controls *aveR* and *ohrB1*, we performed EMSAs to determine whether OhrR binds to promoter regions of other putative targets. OhrR bound specifically only to the promoter region of *pykA2* (encodes a putative pyruvate kinase) and the intergenic region of *tkt2*/*ctaB* (encodes a putative transketolase and protoheme IX farnesyltransferase) (**Figure 7**). *tkt2* and its downstream genes *tal2*, *zwf2*, *opcA2*, and *pgl* have the same transcription direction with very short intergenic regions and may be located in the same transcription unit. The transcription levels of *pykA2*, *ctaB*, *tkt2*, *tal2*, *zwf2*, and *pgl* were higher in DohrR than in WT on both day 2 and day 6, indicating that the genes are under negative control of OhrR.

## Discussion

Our findings demonstrate that OhrR in *S. avermitilis* acts as a repressor specifically in response to organic peroxide stress. OhrB2 is the major organic hydroperoxide resistance protein responsible for such stress. In the absence of organic peroxide stress, the reduced form of OhrR binds to the OhrR box of the *ohrR*–*ohrB2* intergenic region and represses the expression of *ohrB2* and its own. In the presence of such stress, oxidized OhrR dissociates from OhrR box, and *ohrB2* expression is increased by derepression. Similar regulatory roles of OhrR have been reported for *S. coelicolor* and other bacterial taxa (Fuangthong et al., 2001; Panmanee et al., 2002; Oh et al., 2007), indicating a conserved role of OhrR in organic peroxide stress responses. Negatively regulation of *ohrR* itself provides a mechanism for rapidly reduced the expression of major organic hydroperoxide resistance protein in response to disappearance of organic peroxide stress.

Avermectin production in *S. avermitilis* was enhanced when *ohrR* gene was deleted. OhrR binds to two OhrR boxes located upstream of the *aveR* TSS, and represses *aveR* expression. OhrR thus represses avermectin production by controlling the expression of pathway-specific activator gene *aveR*. Several transcription factors have been shown to control avermectin production through direct regulation of *aveR* expression; these include phosphate metabolism regulator PhoP, pseudo γ-butyrolactone receptor homolog AvaR2, GBL receptor AvaR1, and Redox-sensing regulator Rex (Yang et al., 2015; Zhu et al., 2016, 2017; Liu et al., 2017). The present results demonstrate that avermectin production is directly regulated by OhrR. The site 1 of OhrR-binding sites in the *aveR* promoter region is located upstream -35 region (-63 to -51), and the site 2 is also the binding site of Rex which represses *aveR* expression. Therefore, OhrR regulates the expression of *aveR* possibly through blocking RNA polymerase to the promoter or interfering Rex or other regulator to bind to the *aveR* promoter region.

The genes *pykA2* and *ctaB/tkt2* are also directly regulated by OhrR. *pykA2* encodes a putative pyruvate kinase that catalyzes the final step of glycolysis by transferring a phosphate group from phosphoenolpyruvate to adenosine diphosphate, to yield pyruvate and ATP (Ponce et al., 1995). *tkt2* and its downstream genes *tal2*, *zwf2*, *opcA2*, and *pgl* may be located in the same transcription unit. These genes encode putative transketolase, transaldolase, glucose-6-phosphate dehydrogenase, oxppcycle protein, and 6-phosphogluconolactonase of the pentose phosphate (PP) pathway (Sprenger, 1995). *ctaB* encodes a putative protoheme IX farnesyltransferase (CtaB) involved in synthesis of heme containing terminal oxidases of the bacterial respiratory chain (Xu et al., 2016). In cells subjected to organic peroxide stress, oxidized OhrR activates expression of *pykA2* and *ctaB/tkt2-tal2-zwf2*-*opcA2*-*pgl* by derepression, thereby enhancing glycolysis and PP pathway. *Streptomyces* have no glutathione/glutaredoxin system; instead, they use thioredoxin to reduce protein disulfide bonds formed during oxidative stress. The oxidized form of thioredoxin is reduced by thioredoxin reductase, with expenditure of NADPH (Stefankova et al., 2006; Koharyova et al., 2011). Enhancement of PP pathway generates new NADPH, which helps prevent oxidative stress. Intermediate metabolites generated during PP pathway, which include NADPH, ribose-5-phosphate, and erythrose-4-phosphate, are key precursors in the processes of cell growth, DNA biosynthesis, and DNA repair (Zhang et al., 2003). New ATP is produced by glycolysis, and protoheme IX farnesyltransferase overexpression promotes recovery from energy-depleted condition resulting from respiratory chain damage during oxidative stress. Enhancement of glycolysis and PP pathway may likewise stimulate avermectin production in *S. avermitilis*.

In summary, organic peroxide-sensing repressor OhrR functions as a repressor, mediating organic peroxide induction of *ohr* genes through derepression. OhrR represses avermectin production by directly regulating pathway-specific activator gene *aveR.* OhrR also has a negatively regulatory role in glycolysis and PP pathway by controlling the expression of *pykA2* and *ctaB*/*tkt2*-*tal2*-*zwf2*-*opcA2*-*pgl* in *S. avermitilis*.

## Author Contributions

ZC and MS designed the experiments. MS and ML performed the experiments. MS, ML, and ZC analyzed the data and wrote the manuscript. YW, YS, and JL contributed the study materials.

## Conflict of Interest Statement

The authors declare that the research was conducted in the absence of any commercial or financial relationships that could be construed as a potential conflict of interest.
