# Supplementary material for: Organic Peroxide-Sensing Repressor OhrR Regulates Organic Hydroperoxide Stress Resistance and Avermectin Production in Streptomyces avermitilis
Source: Front Microbiol. 2018 Jun 29;9:1398. doi: 10.3389/fmicb.2018.01398 (PMC6034001; doi:10.3389/fmicb.2018.01398)
Supplement: Supplementary file 1 [file Data_Sheet_1.PDF]

Supplemental Materials

Organic peroxide-sensing repressor OhrR regulates organic hydroperoxide stress resistance and avermectin production in *Streptomyces avermitilis*

Meng Sun*[#]*, Mengya Lyu*[#]*, Ying Wen, Yuan Song, Jilun Li, and Zhi Chen*

State Key Laboratory of Agrobiotechnology and Key Laboratory of Soil Microbiology,

Ministry of Agriculture, College of Biological Sciences, China Agricultural University,

Beijing 100193, China

*** Correspondence:**

Zhi Chen

chenzhi@cau.edu.cn

TABLE S1. Primers used in this study.

Purpose	Primer	Sequence (5'--3')[a]
Construction of DohrR mutant		
	OR-up-Fw	CGGAATTCGCTCGATACGCAGGGTGAC(*Eco*RI)
	OR-up-Rev	GCTCTAGAGAGGGAGAAGCAGATCTGGC(*Xba*I)
	OR-dw-Fw	GCTCTAGACTCACGACGGCACTGGAC(*Xba*I)
	OR-dw-Rev	CGGGATCCGCTCTCACGTATCCCGACG(*Bam*HI)
Verification of DohrR mutant		
	OR-V-Fw	GGCTCGACTGGCTCGTCCTAC
	OR-V-Rev	TGGTCCTGCTGGCGGTGTC
	OR-V2-Fw	GGTGATGATGGTGCTGTG
	OR-V2-Rev	CGTGAGGTGGACGTACAC
Complementation of DohrR mutant		
	OR-C-Fw	CGGAATTCCTCTCAAAGGTCGTGTACGG(*EcoR*I)
	OR-C-Rev	CGGGATCCGGAGTTCGTGCAGAAGGG(*Bam*HI)
Construction of His$_6$-tagged OhrR		
	His-ohrR-Fw	CGGAATTCATGAGCGCCATCCCGACC(*Eco*RI)
	His-ohrR-Rev	CCAAGCTTCTGTACGTACGCCGTCCC(*Hin*dIII)
EMSA assay		
	aveD-A1p-Fw	CCGTCCATCCTCTGCACCTG
	aveD-A1p-Rev	GCTGTCGTGGCGATCTACTCC
	aveRp-Fw	GATGGCCTTCTCCTCCGG
	aveRp-Rev	CGTGAGTTCTTCTGGTTTCCG
	cspD3p-Fw	GCGACAGGCGGAGGGAAA
	cspD3p-Rev	ACCACTTCACGGTGCCAGTAG
	cydA1p-Fw	CTCACGCAGGTCTTCCACAC
	cydA1p-Rev	AGCGAGGTCTTCATCACGGG
	fadE3p-Fw	GCGGTCGGTGCTGAGGAG
	fadE3p-Rev	GCGGTCCCAGGCGATGAC
	hrdBp-Fw	CATCGTTGACCACCTATGACC
	hrdBp-Rev	CTCTCGGAACGTTGGAAAAC
	ilvAp-Fw	GAGGCGGTTGAGCAGCAGATAC
	ilvAp-Rev	GTACGTCGTCGAGCGTCACC
	ohrB1p-Fw	GGGAAGAGCTGTGGCGGC
	ohrB1p-Rev	TGGCCTCGGCGACGTACAT
	ohrR-ohrB2p-Fw	GGCAACAGCGGTGTAGAG
	ohrR-ohrB2p-Rev	CGGAGGTAGTTCTCGTCGG
	paaA-Fw	GCTTCCCGTTTCCGCCGTC
	paaA-Rev	CGCTGCTGCTGTCGCCAT

	pykA2p-Fw	CGAACCTCAACGCCAGAACG
	pykA2p-Rev	GCCTCGACCAGTGCCTTGA
	sig7p-Fw	TCTCGCCGACTGGAACTCCTTC
	sig7p-Rev	CGTGCCGCCGGTAGAACTCT
	sig57p-Fw	GCCTGAGCCGTTCCCAGTTC
	sig57p-Rev	GTGCTGCTTCGTTCCAGTCGT
	tkt2-ctaBp-Fw	GGGTGGGCTCGTGTTGGGA
	tkt2-ctaBp-Rev	TCTGTGGTGGTCGGCTTGGT
5' RACE		
	oligo(dT)-anchor primer	GACCACGCGTATCGATGTCGACTTTTTTTTTTTTTTTT
	sp1-ohrR	CTCCGTGAGGTGGACGTAC
	sp2-ohrR	GGTACTGGGGGTAGGTGAGCC
	sp3-ohrR	CGTTGAGGGAGAAGCAGATCTG
	sp1-ohrB2	CCGAAGCCCTCACCCTGC
	sp2-ohrB2	GTAACCGCCGGCGAACAG
	sp3-ohrB2	GACCGTTGCCGCCCAGCTC
qRT-PCR		
	aveA1-QP-Fw	CGGACAGGACTACGCACTTC
	aveA1-QP-Rev	ACGAGATACGACCGGAGATG
	aveR-QP-Fw	CAGAAGAACTCACGCTCGTC
	aveR-QP-Rev	ACTCTTTCCACAGCCCATTC
	ctaB-QP-Fw	GGTGCTCATCGGCTGGTCCT
	ctaB-QP-Rev	TAGTGCGGCGGCGTCCAG
	hrdB-QP-Fw	TACTGCGCAGCCTCAACCAG
	hrdB-QP-Rev	GCCGATCTGCTTGAGGTAGTC
	ohrB1-QP-Fw	CTTCCACAACGCGCTGGTG
	ohrB1-QP-Rev	GACACGCTGAGGGCGACC
	ohrB2-QP-Fw	AGCGCCCTCGGTCTCGTC
	ohrB2-QP-Rev	AGCCCTCACCCTGCTTGC
	ohrR-QP-Fw-1	ATGAGCGCCATCCCGAC
	ohrR-QP-Rev-1	GAGGGAGAAGCAGATCTGGC
	pgl-QP-Fw	CGGGACGCCATCGACTGGG
	pgl-QP-Rev	CTCACGGGCCTGGGTGTCAT
	pykA2-QP-Fw	GGAGTCCTCGGCGACCTTCAA
	pykA2-QP-Rev	CCTTCCTCGACGGTGATGGTGA
	tal2-QP-Fw	GGCAACCTCGCCGAACTGATC
	tal2-QP-Rev	AACCGTCGCCCTGAGAGATGG
	tkt2-QP-Fw	CAGAAGGTGATGCGGCACGAC
	tkt2-QP-Rev	GTAGAGGGTCAGGGACGAGTGG
	zwf2-QP-Fw	TGATGCCAGCCGTGTACGATCT
	zwf2-QP-Rev	GTGGACGACCTGGGCGAAGT
Footprinting assay		
	aveR-FAM-Fw	CCTCTGGACCCTTGCTCG

aveR-FAM-Rev	CGTGAGTTCTTCTGGTTTCCG
ohrRB2-FAM-Fw	GAACAGCTGCTCCGGGTTG
ohrRB2-FAM-Rev	AGATGCTCGCCGACCTTC

[a]The restriction enzyme sites are underlined.

FIGURE S1

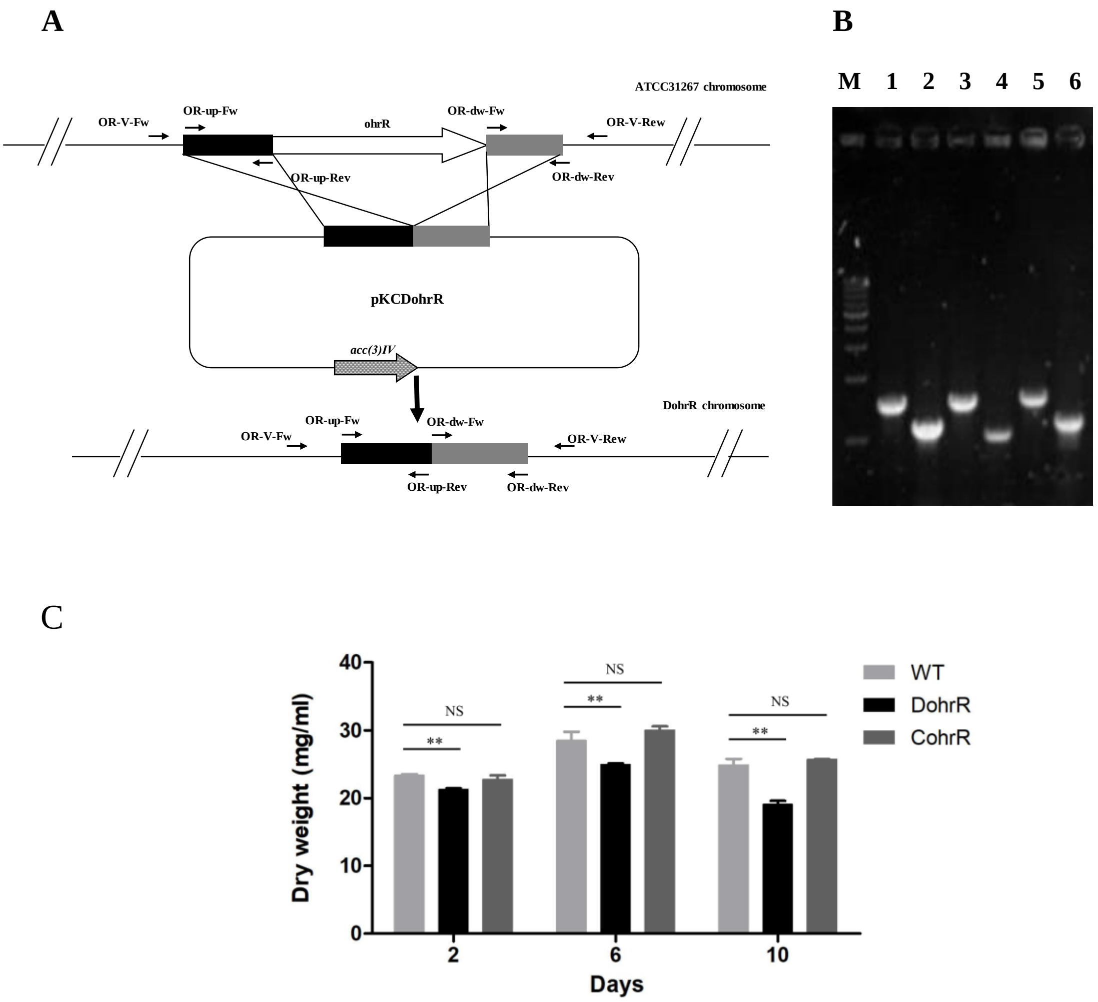

FIGURE S1. Strategy for deletion of *ohrR* gene and PCR analysis of *ohrR*-deletion mutant. (A) Large white arrow: gene and its direction. Small black arrows: primers used for cloning of exchange regions and confirmation of gene deletions, as described in M&M. Double-crossover recombination led to *ohrR* deletion. (B) PCR analysis to confirm *ohrR* deletion in DohrR mutant. Agarose gel electrophoresis of PCR products from WT and DohrR using primer pairs OR-V-Fw/ OR-dw-Rev (lanes 1, 2), OR-up-Fw/ OR-V-Rev (lanes 3, 4), and OR-V-Fw/ OR-V-Rev (lanes 5, 6). Lane M: DL2000 marker. Lanes 1, 3, 5: PCR products from WT. Lanes 2, 4, 6: PCR products from DohrR. (C) Dry weight of WT, DohrR and CohrR in FM-I for 2, 6 and 10 days. NS, not significant ; **, P < 0.01 (Student's t-test).

FIGURE S2

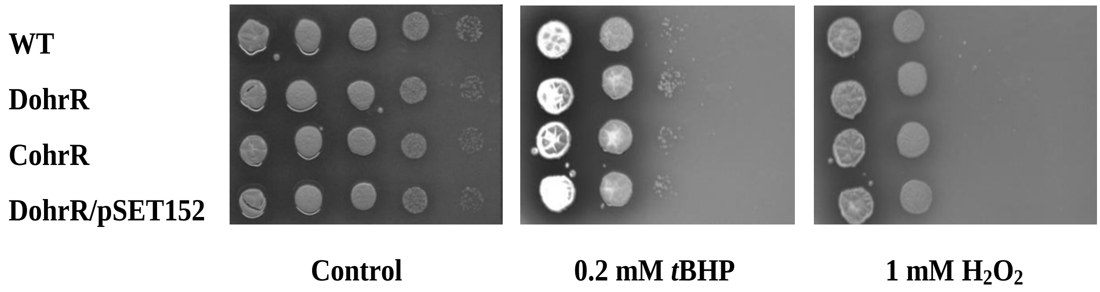

FIGURE S2. Growth of WT, DohrR, and CohrR on YMS with 0.2 mM *t*BHP or 1 mM H_2O_2. Serially diluted spores were spotted on YMS plates with or without oxidants, and incubated for 3 days at 28 °C. DohrR/pSET152: empty plasmid-containing control.

FIGURE S3

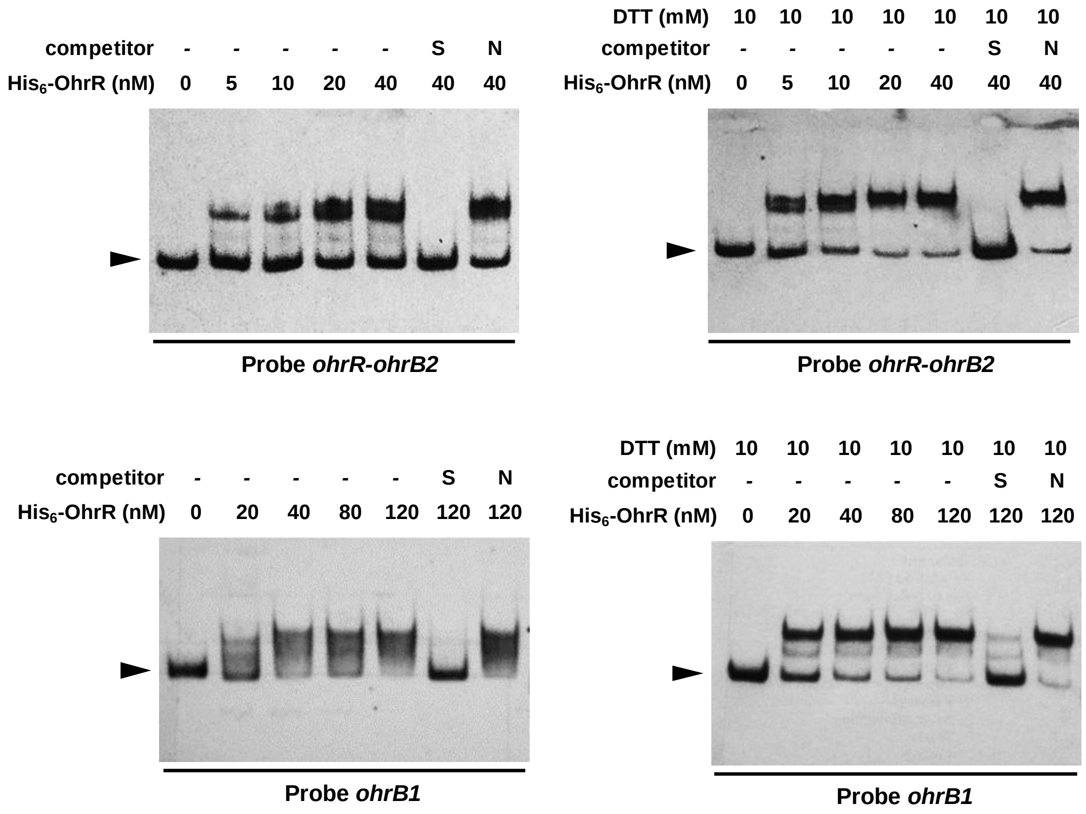

FIGURE S3. Effects of DTT on *in vitro* binding of His_6-OhrR to *ohrR-ohrB2* intergenic region and *ohrB1* promoter region. Top: concentrations of His_6-OhrR and DTT.
